# The Epstein–Barr virus nuclear antigen-1 upregulates the cellular antioxidant defense to enable B-cell growth transformation and immortalization

**DOI:** 10.1038/s41388-019-1003-3

**Published:** 2019-09-11

**Authors:** Jiayu Wang, Noemi Nagy, Maria G. Masucci

**Affiliations:** Department of Cell and Molecular Biology, Karolinska Institutet, Stockholm, Sweden

**Keywords:** Mechanisms of disease, Tumour virus infections

## Abstract

Epstein–Barr virus (EBV) immortalizes human B-lymphocytes and is implicated in the pathogenesis of lymphoid and epithelial cell malignancies. The EBV nuclear antigen (EBNA)-1 induces the accumulation of reactive oxygen species (ROS), which enables B-cell immortalization but causes oxidative DNA damage and triggers antiproliferative DNA damage responses. By comparing pairs of EBV-negative and -positive tumor cell lines we found that, while associated with the accumulation of oxidized nucleotides, EBV carriage promotes the concomitant activation of oxo-dNTP sanitization and purging pathways, including upregulation of the nucleoside triphosphatase mut-T homolog 1 (MTH1) and the DNA glycosylases 8-oxoguanine-glycosylase-1 (OGG1) and mut-Y homolog (MUTYH). Expression of EBNA1 was reversibly associated with transcriptional activation of this cellular response. DNA damage and apoptosis were preferentially induced in EBNA1-positive cell lines by treatment with MTH1 inhibitors, suggesting that virus carriage is linked to enhanced vulnerability to oxidative stress. MTH1, OGG1, and MUTYH were upregulated upon EBV infection in primary B-cells and treatment with MTH1 inhibitors prevented B-cell immortalization. These findings highlight an important role of the cellular antioxidant response in sustaining EBV infection, and suggests that targeting this cellular defense may offer a novel approach to antiviral therapy and could reduce the burden of EBV associated cancer.

## Introduction

Chronic infections by DNA tumor viruses, including oncogenic papilloma (HPV) and polyoma (HPyV) viruses, hepatitis B virus (HBV) and the herpesviruses Epstein–Barr virus (EBV) and Kaposi sarcoma virus (KSHV), account for approximately ten percent of all human malignancies worldwide [1]. A characteristic feature of the virus-induced cancers is the long period, often years or decades, that separate primary infection from clinical manifestation, suggesting that infection acts as the initiating event while the accumulation of genetic and epigenetic alteration is required for progression to full malignancy [2]. Viral oncogenesis can be regarded as the failure of host controls to restrain viral activities that are primarily devoted to promote efficient replication and spread. A corollary of this scenario is the continuous expression in tumor cells of viral products, including proteins and noncoding RNAs, that drive infection by remodeling cellular functions, such as DNA replication, apoptosis, and cell metabolism, whose deregulation constitutes the hallmark of malignancy.

Malignant transformation is often associated with elevated intracellular levels of reactive oxygen species (ROS). Low levels of ROS are required for intracellular signaling while, at high levels, ROS cause irreversible damage to lipids, proteins, and DNA, and may contribute to the genomic instability that characterize many tumor types [3–5]. A major oxidized base lesion generated by ROS is 8-oxodG that is stable and highly mutagenic because it can pair with cytosine as well as adenine, causing G to T or A to C transversion mutations [6]. Thus, the accumulation of 8-oxodG has been widely used as a biomarker for oxidative stress and carcinogenesis [7]. Viral products are known to drive the establishment of an oxidative environment in the infected cells [8–11]. A noticeable example is the capacity of the EBV nuclear antingen-1 (EBNA1), the only viral antigen consistently expressed in all EBV carrying cells, to upregulate the catalytic subunit of the NADPH oxidase NOX2 [12]. Upregulation of NOX2 correlates with the accumulation of intracellular ROS and consequent induction of chromosomal instability and telomere dysfunction in EBV carrying malignant cells [13]. The need for high levels of ROS is a defining feature of EBV infection since treatment with ROS scavengers severely impairs the growth transformation of B-lymphocytes [14], which prevents the establishment of a reservoir of latently infected cells from which the virus may reactivate and spread to new susceptible host [15].

The oxidative DNA damage caused by excessive intracellular levels of ROS triggers a variety of cell intrinsic antiproliferative and antitumor responses such as cell cycle arrest, cell senescence, and apoptosis [16]. To avoid the harmful effects of ROS, many tumors develop adaptive responses, including the upregulation of protective redox buffering systems [17], the activation of sanitization pathways that prevent the incorporation of damaged nucleotides into newly synthesized DNA [18], and the activation of DNA repair pathways such as nucleotide and base excision repair (NER and BER) that purge DNA from oxidated bases to restore nucleic acid integrity [19]. It has been argued that the reliance on these protective mechanisms may render malignant cells particularly vulnerable to therapeutic interventions that alter the cellular redox balance or specifically target the repair of oxidated DNA [20].

In this investigation we have explored the mechanisms by which EBV infected cells overcome the antiproliferative effects of the elevated levels of ROS induced by EBNA1. By comparing pairs of EBV-negative and -positive cell lines derived from lymphoid and epithelial cell malignancies, we found that EBV carriage is consistently associated with upregulation of the nucleoside triphosphatase mut-T homolog 1 (MTH1) that sanitizes oxidized purines from the free nucleotide pool, and components of the BER and NER pathways, including the glycosylases 8-Oxoguanine glycosylase (OGG1) and mut-Y homolog (MUTYH) that purge oxidized bases from DNA. Expression of EBNA1 from a tetracycline-regulated promoter induced a reversible dose-dependent increase of MTH1, OGG1, and MUTYH confirming that the viral protein is directly involved in driving their expression. Treatment with the MTH1 inhibitors TH588 and (S)-Crizotinib selectively induced DNA damage and apoptosis in EBV-positive cell lines and prevented the establishment of EBV transformed lymphoblastoid cell lines from freshly infected B-lymphocytes, suggesting that activation of the cellular defense against oxidative stress plays a central role in promoting the growth transformation and survival of EBV infected cells.

## Results

### EBV carriage is associated with the accumulation of oxidated nucleotides

**Fig. 1:**
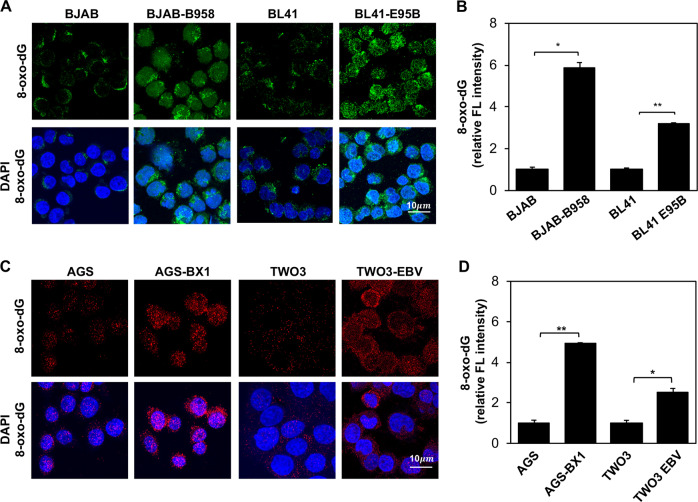
EBV carriage is associated with the accumulation of 8-oxodG. **a** Pairs of EBV-negative and -positive B-cell lymphoma lines were subjected to immunofluorescence staining with antibody against 8-oxodG (green) and the nuclei were stained with DAPI (blue). Representative micrographs illustrating the expression of 8-oxodG in the BJAB and BL41 cell pairs. **b** Quantification of 8-oxodG specific fluorescence in three independent experiments. Relative FL intensity is the ratio between FL intensity in EBV positive and negative cells. **c** 8-oxodG fluorescence was assayed in pairs of EBV-negative and -positive epithelial cell lines. Representative micrographs illustrating the expression of 8-oxodG (red). The nuclei were stained with DAPI (blue). **d** Quantification of the 8-oxodG specific fluorescence in three independent experiments. Significance was calculated using Student T-test, *P *<* 0.05, **P *<* 0.01

**Fig. 2:**
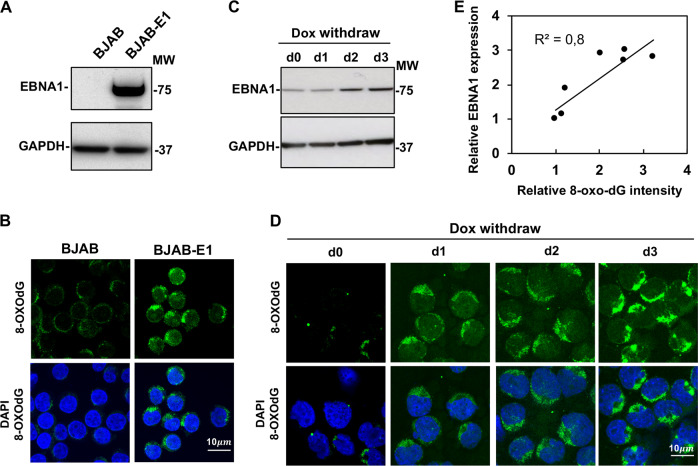
The accumulation of 8-oxodG is dependent on EBNA1 expression. The accumulation of 8-oxodG was investigated in BJAB sublines with stable or inducible EBNA1 expression. **a** Representative western blots illustrating the expression of EBNA1 in BJAB and the stably transfected subline BJAB-E1. GAPDH was used as loading control. **b** BJAB and BJAB-E1 cells were subjected to immunofluorescence staining with antibody against 8-oxodG (green) and the nuclei were stained with DAPI (blue). Micrographs are representative of three independent experiments. **c** Representative western blots illustrating the upregulation of EBNA1 in BJAB-tTAE1 after removal doxycycline. GAPDH was used as loading control. **d** Representative micrographs illustrating the increase of 8-oxodG in BJAB-tTAE1 after induction of EBNA1 by removal of doxycycline. Micrographs are representative of two individual experiments. **e** Regression analysis of the relationship between the expression levels of EBNA1 and 8-oxodG. The data of two doxycycline withdrawal experiments were plotted

Previous studies have shown EBV carriage promotes the accumulation of high levels of ROS, which induces oxidative DNA damage and activates the DDR. 8-Oxoguanine (8-oxodG) is the major oxidized base in the free nucleotide pool and in DNA [21], and is widely used as a biomarker for oxidative stress [18]. To clarify the relationship between EBV carriage and the accumulation of oxidated nucleotides, the presence of 8-oxodG was detected by immunofluorescence in EBV-negative cell lines derived from B-cell lymphoma (BJAB, BL41), nasopharyngeal carcinoma (TWO3), or gastric cancer (AGS) and in their EBV converted sublines obtained by in vitro infection with transforming stains of EBV. EBV carriage was confirmed in the converted sublines: BL41-E95B, BJAB-B958, AGS-BX1, and TWO3-EBV, by probing western blots of total cell lysates with EBNA1 specific antibodies. Representative micrographs illustrating the elevated expression of 8-oxodG in EBV carrying cells are shown in Fig. 1a, c. Quantification of fluorescence intensity in three independent experiment (Fig. 1b, d) confirmed a significant increase of fluorescence intensity in all EBV converted sublines compared with the EBV-negative parentals, supporting the conclusion that EBV carriage is associated with a shift of the oxidative balance leading to the accumulation of oxidated nucleotides. Of note, a comparable increase was observed in both B-cell and epithelial cell lines suggesting that the effect is not cell type specific. In line with our previous finding that EBNA1 is responsible for the accumulation of ROS via upregulation of NOX2 [12], we found that stable expression of EBNA1 in the BJAB-E1 transfected cell line (Fig. 2a), was sufficient for a strong increase of 8-oxodG specific fluorescence (Fig. 2b). Furthermore, 8-oxodG fluorescence was significantly increased in parallel with upregulation of EBNA1 upon withdrawal of doxycycline in BJAB-tTAE1 cell lines that carries the viral gene under control of a tet-off regulated promoter (Fig. 2c, d). Plotting the intensity of the EBNA1 specific band versus the intensity of 8-oxodG florescence confirmed a significant positive correlation between the levels of EBNA1 expression and the extent of 8-oxodG accumulation (Fig. 2e).

### EBNA1 promotes the upregulation of antioxidant defense systems

**Fig. 3:**
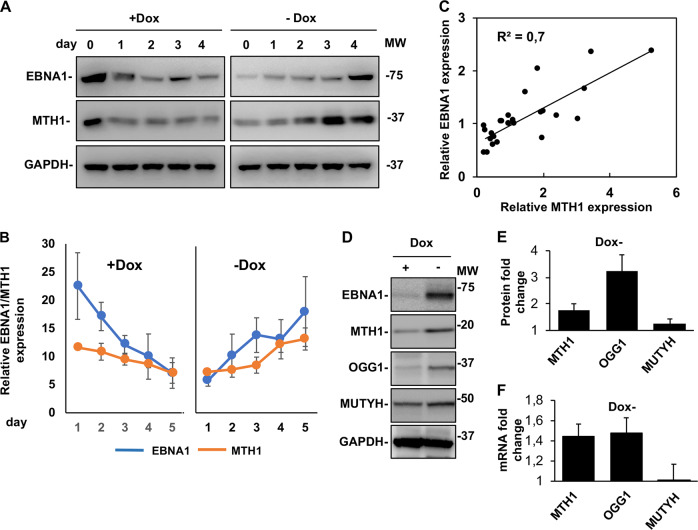
EBNA1 expression is associated with upregulation of MTH1 and oxidative damage repair pathways. The expression of MTH1, OGG1, and MUTYH was investigated by western blots and qPCR in BJAB-tTAE1 cells upon addition and withdrawal of doxycycline. **a** Representative western blots illustrating the correlation between the reversible down- and upregulation of MTH1 and EBNA1 in BJAB-tTAE1 cells upon addition and withdrawal of doxycycline. GAPDH was used as loading control. **b** Densitometry quantification of MTH1 and EBNA1 expression in BJAB-tTAE1 cells. The mean intensity of the MTH1 and EBNA1 specific bands relative to GAPDH in three independent experiments is shown in the figure. **c** Regression analysis of the relationship between expression levels of MTH1 and EBNA1. The data from three independent experiments were used for the plot. **d** Representative western blots illustrating the expression of MTH1, MUTYH, and OGG1 in BJAB-tTAE1 cells cultured for two weeks in the presence or absence of doxycycline. **e** Fold change is the ratio between the intensity of the specific band in cells cultured without or with doxycycline. The mean ± SD of four independent experiments is shown. **f** qPCR analysis of the levels of MTH1, OGG1, and MUTYH transcripts in BJAB-tTAE1 cells cultured for 2 weeks in the presence or absence of doxycycline. The mean ± SE of the fold change in six independent experiments each performed in triplicate is shown

The capacity of EBNA1 to induce oxidative stress suggests that EBNA1 or other viral products may counteract the antiproliferative effects of oxidative DNA damage. An effective defense strategy would be to limit the damage by sanitizing oxidized nucleotides, to prevent their incorporation into nascent DNA, or purging the oxidated DNA, via activation of NER and BER pathways. The MTH1 pyrophosphatase plays a key role in sanitization of the free nucleotide pool by converting 8-oxo-7,8dihydro-2′deoxoguanosine triphosphate (8-oxodGTP) into 8-oxodGDP that cannot be incorporated into DNA [22]. In order to assess whether EBNA1 activates this pathway, the expression of MTH1 was investigated in the BJAB-tTAE1 cells cultured in the presence or absence of doxycycline. A time course of EBNA1 expression confirmed that while the viral protein is expressed at high levels when the cells are cultured in the absence of doxycycline, the expression decreased upon addition of doxycycline to return at normal levels after doxycycline withdrawal (Fig. 3a). In accordance with our previous findings [12], this was accompanied by a corresponding reversible increase of ROS levels (Supplementary Fig. S1). Probing of western blots with specific antibodies revealed that changes in the expression of EBNA1 were paralleled by changes in the intensity of the MTH1 specific band (Fig. 3a, b). The upregulation of MTH1 expression was dependent in the expression of EBNA1 since treatment with doxycycline had no effect on the expression of MTH1 in the control BJAB-tTA cell line (Supplementary Fig. S2). The relationship between EBNA1 and MTH1 was further supported by regression analysis that confirmed a significant positive correlation between the expression levels of the two proteins (Fig. 3c).

Oxidized dNTPs that are incorporated into DNA are removed by DNA glycosylases, such as OGG1 or MUTYH, to initiate BER, while the scaffold protein XRCC1 interacts with DNA polymerase beta and DNA ligase III to complete the repair process [23]. To examine whether this pathway is involved in the rescue of oxidative stress induced by EBNA1, we assessed the expression of MUTYH and OGG1 in BJAB-tTAE1 cultured in the presence or absence of doxycycline. All three proteins were consistently upregulated in the EBNA1 expressing cells with stronger effect reproducibly observed for OGG1 followed by MTH1 and by a small but reproducible upregulation of MUTYH (Fig. 3d, e). In accordance with the capacity of EBNA1 to regulate the transcription of a broad variety of cellular genes [24], qPCR analysis of mRNA isolated from cells cultured in the presence or absence of doxycycline revealed reproducible upregulation of the MTH1 and OGG1 transcripts in EBNA1 expressing cells while only a minor effect was observed for MUTYH (Fig. 3f).

**Fig. 4:**
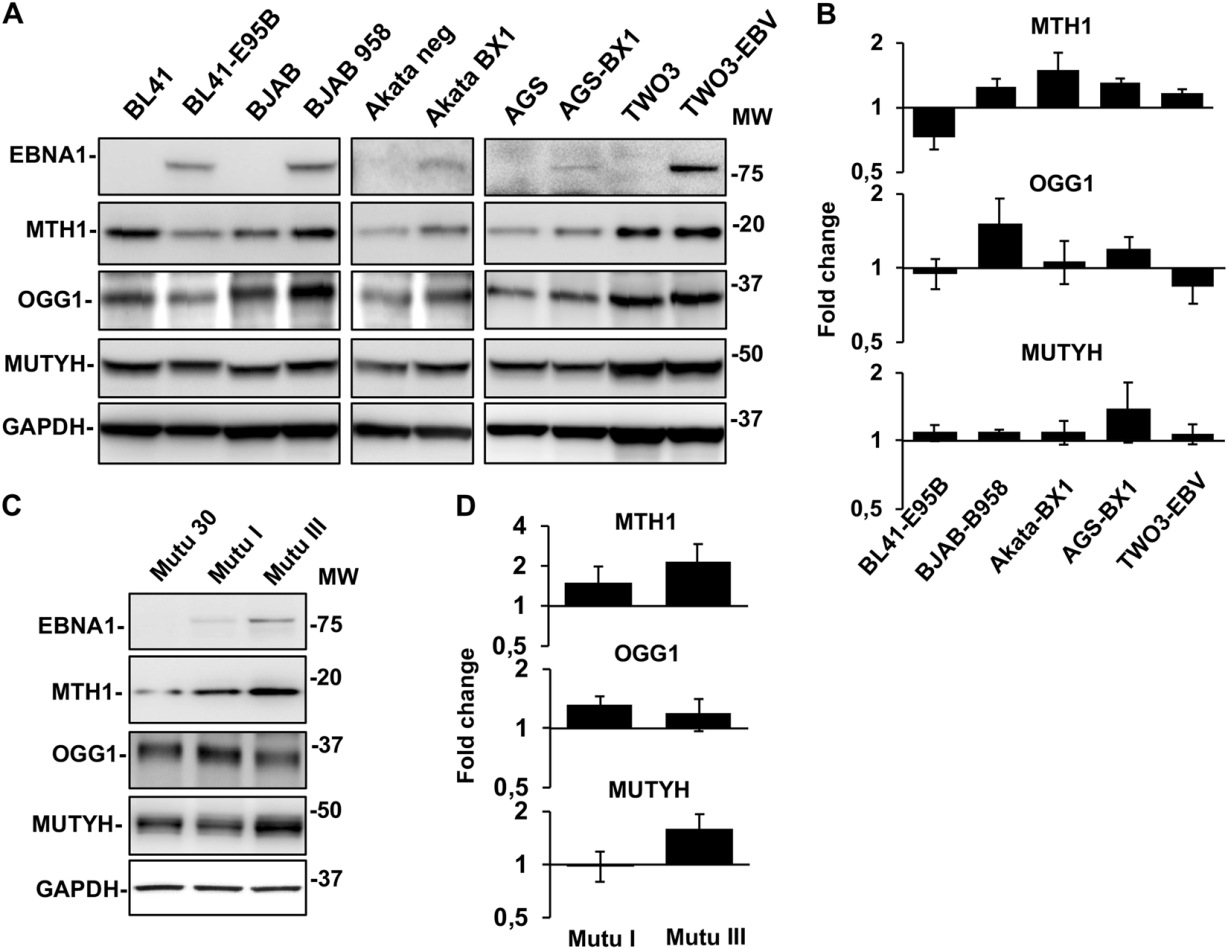
EBNA1 promotes the upregulation of oxidative DNA damage repair pathways in EBV converted cell lines and EBV positive BLs. **a** Representative western blots illustrating the expression of MTH1, OGG1, and MUTYH in pairs of EBV-negative and -positive cell lines. GAPDH was used as loading control. **b** Densitometry quantification of the specific bands. The intensity of the specific band in EBV positive cells relative the EBV-negative parental is shown. Mean ± SE of four independent experiments. **c** Representative western blots illustrating the expression of EBNA1, MTH1, OGG1, and MUTYH in the Mutu cell lines. **d** Densitometry quantification of expression in the EBV positive cell lines relative to the EBV-negative Mutu-30. Mean ± SE of four independent experiments

In order to assess whether EBNA1 activates these pathways also under physiological levels of expression and in originally EBV positive BLs, the expression of MTH1, OGG1, and MUTYH was compared in pairs of EBV-negative and converted cell lines and in a panel of EBV positive and negative sublines of the Mutu BL (Fig. 4). As illustrated by representative western blot shown in Fig. 4a and quantification of the specific bands in four independent experiments (Fig. 4b), a slight increase of MTH1 was detected in four of the five EBV converted sublines compared with their EBV-negative parental lines while two independently established EBV converted sublines of BL41 showed decreased MTH1 expression (Fig. 3a, b and not shown). Different patterns of expression were observed for OGG1 and MUTYH. A small increase of OGG1 was detected in the BJAB-B958 and AGS-BX1, where MUTYH was also increased, while only minor changes were detected in the remaining cell lines. Two sublines of the originally EBV positive BL Mutu expressed MTH1, OGG1, and MUTYH at consistently higher levels compared with the Mutu-30 subline that has lost the virial genome. In line with the capacity of EBNA1 to induce oxidative stress and regulate the antioxidant pathways, the highest levels of MTH1 and MUTYH were detected in the Mutu III subline that expressed a type III latency program with higher EBNA1 expression.

### MTH1 inhibitors induce apoptosis and DNA damage in EBNA1 positive cells

**Fig. 5:**
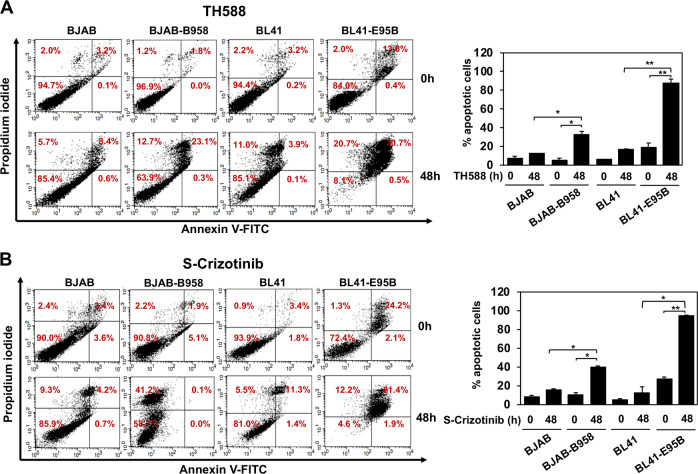
Small molecule inhibitors of MTH1 induce apoptosis in EBV positive cells. **a** Pairs of EBV-negative and -positive cell lines were treated with 10 µM of the MTH1 inhibitor TH588 for 48 h. Apoptosis was measured by Annexin V and PI staining. Bar graph depicts the percentage of apoptotic cells. Data are shown as the mean ± SD two independent experiments. *P *<* 0.05, **P *<* 0.01. **b** Pairs of EBV-negative and -positive cell lines were treated with 5 µM of the MTH1 inhibitor S-Crizotinib for 48 h and apoptosis was measured using Annexin-V and PI staining. Bar graph depicts the percentage of apoptotic cells. Data are shown as the mean ± SD of two independent experiments. *P *<* 0.05, **P *<* 0.01

Collectively, these findings suggest that the activation of antioxidant pathways may be critical for the survival of EBV carrying cells. In order to test this possibility, we compared the panel of EBV-negative and -positive cell lines and transfectants with stable or inducible EBNA1 expression for their sensitivity to treatment with the MTH1 small molecule inhibitors TH588 and S-Crizotinib [25, 26]. PI/Annexin-V staining of the BJAB/BJAB-B958 and BL41/BL41-E95B cell pairs treated for 48 h with 10 μM TH588 (Fig. 5a, b) or 5 μM S-Crizotinib (Fig. 5c, d) revealed that, while the treatment had a small or no effect on the survival of the EBV-negative parental, both inhibitors induced a significant increase of apoptosis in the EBV converted sublines. Interestingly, the effect was stronger in the BL41-E95B cells line that expresses relatively lower levels of MTH1 (Fig. 4a, b), suggesting that these cells are particularly dependent on MTH1 activity for their survival. The enhanced sensitivity of EBV carrying cells was further confirmed in the extended panel of paired cell lines that included both B-cells and epithelial cells. As a rule, the converted lines were more sensitive to both inhibitors compared with their EBV-negative parentals (Table 1). Exceptions were Akata-BX1 that did not respond to S-Crizotinib and the TWO3/TWO3-EBV cell pair, where the EBV-negative parental was highly sensitive to TH588. Conceivably, cell-specific adaptation strategies established during long term in vitro propagation may account for these discrepancies. In line with this possibility, the upregulation of MTH1 induced by stable (BJAB-E1) or inducible expression of EBNA1 (BJAB-tTAE1) was consistently associated with enhanced sensitivity to both inhibitors (Table 1). The sensitivity of the BJAB-tTAE1 cell lines could be reversed in repeated cycles of doxycycline treatment and withdrawal, which did not affect significantly the response of control BJAB-tTA (Fig. S3), confirming that EBNA1 is directly responsible for the enhanced sensitivity to inhibition of MTH1. The effect of the inhibitor was also independent of significant changes in the expression of viral genes as confirmed by probing western blot of lysates from treated cells with antibodies specific for the latency antigens EBNA1 and LMP1 and the lytic immediate-early protein BZLF1 (Fig. S4).

**Table 1:** EBV carriage renders the cells more sensitive to inhibition of MTH1

% Apoptotic cells^a^					Fold change^f^	
Cell lines	EBV^b^	Control	TH588^c^	S-crizotinib^d^	TH588	S-crizotinib
BJAB	−	7.7 ± 1.7	12.2 ± 0.1	15.6 ± 1.4	1.6	2.0
BJAB-B958	+	8.1 ± 4.1	32.7 ± 3.4	39.9 ± 1.4	4.0	4.9
BL41	−	5.4 ± 1.0	19.3 ± 4.4	22.9 ± 4.7	3.6	4.3
BL41-E95B	+	14.8 ± 5.5	88.0 ± 4.0	94.5 ± 0.9	6.0	6.4
Akata	−	4.8 ± 1.1	30.3 ± 2.3	11.7 ± 3.8	6.3	2.4
Akata-BX1	+	3.2 ± 1.5	51.9 ± 2.9	7.0 ± 1.2	16.4	2.2
AGS	−	1.6 ± 0.2	14.3 ± 2.7	3.8 ± 0.3	8.9	2.4
AGS-BX1	+	1.7 ± 0.8	26.5 ± 0.9	11.2 ± 0.5	15.3	6.5
TWO3	−	9.8 ± 1.7	27.6 ± 4.6	19.2 ± 2.4	2.8	2.0
TWO3-EBV	+	12.5 ± 4.5	25.6 ± 1.7	54.7 ± 7.0	2.0	4.4
	eEBNA1					
BJAB	−	7.7 ± 1.7	12.2 ± 0.1	15.6 ± 1.4	1.6	2.0
BJAB-EBNA1	+	4.8 ± 1.0	31.4 ± 2.8	21.7 ± 2.5	6.5	4.5
BJAB-tTAE1 Dox+	−	9.0 ± 2.5	19.6 ± 0.2	23.8 ± 1.0	2.2	2.7
BJAB-tTAE1 Dox-	+	9.7 ± 2.3	40.4 ± 1.2	48.1 ± 0.8	4.2	5.0

Values corresponding to ≥ 50% increased apoptosis in EBV/EBNA1 positive cells are underlined

^a^Apoptosis was measured by staining with PI/Annexin-V and flow cytometry analysis. The mean ± SD positive cells in two independent experiments is shown

^b^EBV carriage was in all cases confirmed by detection of EBNA1

^c^The cells were treated with 10 μM TH588 for 48 h

^d^The cells were treated with 5 μM S-Crizotinib for 48 h

^e^EBNA1 expression was confirmed by western blot analysis

^f^ Fold change was calculated as the ratio between the mean % apoptotic cells in control and treated cells

**Fig. 6:**
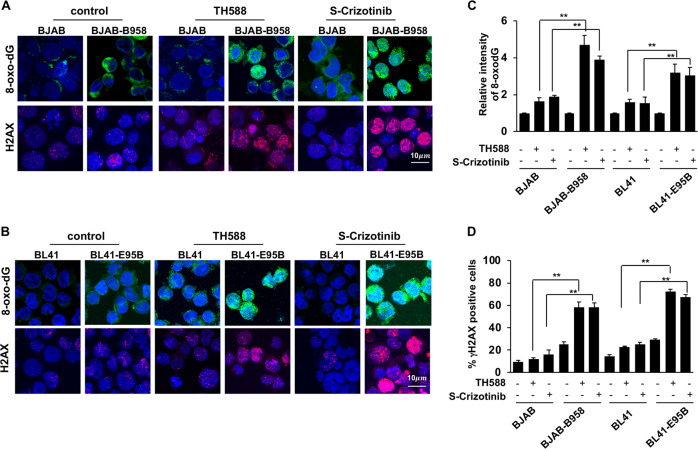
Inhibition of MTH1 is associated with increased levels of oxidative DNA damage in EBV positive cells. Pairs of EBV-negative and -positive cells were treated with TH588 (10 µM) or S-Crizotinib (5 µM) for 24 h followed by immunofluorescence staining with antibodies against 8-oxodG (green) or γH2AX (red) and the nuclei were stained with DAPI (blue). **a** Representative microphotographs illustrating the expression of 8-oxodG and γH2AX in the untreated and treated BJAB/BJAB-B958 cell pair. **b** Representative microphotographs illustrating the expression of 8-oxodG and γH2AX in the untreated and treated BL41/BL41-E95B cell pair. **c** Quantification of the 8-oxoG specific fluorescence. Data are shown as the mean the ± SE of three independent experiments. **P *<* 0.01. **d** Percentage of γH2AX positive cells. Data are shown as the mean ± SE from three independent experiments. **P *<* 0.01

In order to gain insight on the mechanism by which MTH1 inhibitors preferentially induce the apoptosis of EBV positive cells, the expression levels of 8-oxodG and the DDR activation marker γH2AX were monitored in the BJAB/BJAB-B958 and BL41/BL41-E95B cell pairs treated for 48 h with TH588 or S-Crizotinib (Fig. 6a, b). Both inhibitors induced a significantly stronger increase of 8-oxodG in the EBV positive cell lines (Fig. 6a, b upper panels and Fig. 6c). In accordance with previous findings [12], a higher number of γH2AX positive cells was detected in the EBV converted cell lines compared with the EBV-negative parentals. However, while treatment with MTH1 inhibitors has only a small effect in the EBV-negative cells, EBV carriage was associated with a highly significant and reproducible three- to four-fold increase in the number of γH2AX positive cells (Fig. 6a, b lower panels and Fig. 6d). Collectively these findings suggest that due to their enhanced oxidative burden EBV carrying cell lines are remarkably dependent on the function of cellular antioxidant pathways.

### Upregulation of MTH1 counteracts EBV-induced oxidative stress in primary EBV infected cells

**Fig. 7:**
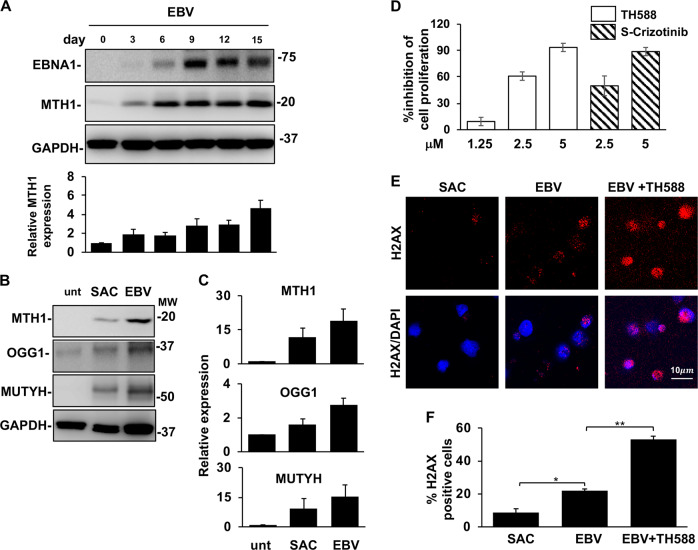
The antioxidant pathways are activated during EBV infection and are required for growth transformation. Freshly isolated B-lymphocytes infected with the transforming B95-8 strain of EBV were cultured for up to 2 weeks in the presence or absence of MTH1 inhibitors. Protein expression was monitored by western blots, cell proliferation and activation of the DDR were assessed by ^3^H-Thy incorporation and staining for γH2AX, respectively. **a** representative western blots illustrating the parallel increase of MTH1 and EBNA1 expression following EBV infection and mean ± SE of the intensity of the MTH1 specific band in five independent experiments. **b** Representative western blots illustrating the expression of MTH1, MUTYH and OGG1 in ex vivo untreated B-cell and freshly EBV infected and SAC induced B blasts cultured for comparable times and showing similar levels of cell proliferation. **c** Quantification of the specific bands. Relative expression is the ration between the intensity in the treated cells versus freshly harvested cells. The mean ± SE of three to four independent experiments is shown. **d** Inhibition of MTH1 prevents the establishment of EBV transformed lymphoblastoid cell lines. ^3^H-Thy incorporation was measured after culture of freshly EBV infected B-lymphocytes in the presence of the indicated amounts of MTH1 inhibitors. Depending on the condition of the cultures harvesting was done after ten to fifteen days. **e** Inhibition of MTH1 strongly enhances the induction of DNA damage in freshly EBV infected cells. DNA damage was detected by γH2AX staining. **f** Mean ± SE of the % γH2AX positive cells in three independent experiments. *P *<* 0.05; **P *<* 0.01

In the next set of experiments, we sought to gain insight on the contribution of antioxidant pathways to the establishment of EBV latency in normal B-lymphocytes. To this end, we first monitored the expression of MTH1 in primary B-cells infected with the transforming B95-8 strain of EBV. As illustrated by the representative blots and densitometry analysis of five independent experiments shown in Fig. 7a, EBV infection was associated with progressive increase of both EBNA1 and MTH1 expression, starting between day 3 and 6 when the peak of ROS begins to subside [14] and culminating after ~2 weeks when immortalized lymphoblastoid cell lines became established. We then compared the expression of MTH1, MUTYH, and OGG1 in proliferating EBV infected cells and B-cell blasts induced by mitogen stimulation. Upregulation of the antioxidant pathways was observed in both types of proliferating cells but the effect was approximately twofold stronger following EBV infection (Fig. 7b, c). Finally, we tested whether interference with the antioxidant balance by inhibition of MTH1 would compromise the establishment of EBV transformed lymphoblastoid cell lines. Freshly EBV infected B-lymphocytes were cultured for 2 weeks in the presence of increasing concentrations of TH588 or S-Crizotinib and cell proliferation measured by tritiated thymidine (^3^H-Thy) incorporation was compared with that of infected cells cultured in the absence of the inhibitors. Both inhibitors cause a dose-dependent reduction of ^3^H-Thy incorporation, with almost complete inhibition of cell proliferation and failure to establish autonomously growing LCLs at 5 μM concentration (Fig. 7d). In line with the capacity of the inhibitors to induce apoptosis and oxidative DNA damage in EBV-positive cell lines, the antiproliferative effect of TH588 on freshly infected B-lymphocytes was accompanied by a highly significant threefold increase of γH2AX positive cells (Fig. 7e, f).

## Discussion

Infection by DNA tumor viruses often occurs in an oxidative environment that enables the expression of growth promoting viral and cellular genes but also causes DNA damage and triggers cell intrinsic antiproliferative responses [27]. Here we have shown that the EBV encoded oncoprotein EBNA1 counteracts this cellular defense by upregulating dNTP sanitization and purging pathways that reset the oxidative burden to a level compatible with cell survival and proliferation. This virus-driven adaptation renders EBV infected cells particularly sensitive to disturbance of the redox status, suggesting that targeting the cellular antioxidant defense may be an effective strategy for interfering with infection and containing the burden of EBV associated cancer.

EBNA1 is the only viral protein regularly detected in all types of proliferating EBV-infected cells and EBV associated malignancies. In addition to its essential role in viral episome maintenance and regulation of viral promoters [28], the interaction of EBNA1 with cellular chromatin is associated with changes of chromatin organization and transcriptional regulation of a broad array of cellular genes [24, 29]. Relevant to this investigation is the previous finding that EBNA1 transcriptionally activates the catalytic subunit of the NADPH oxidase NOX2 [12], which correlates with accumulation of ROS, induction of DNA damage and activation of the DDR. This is at least partially responsible for the extensive cell death observed during the early phase of EBV-induced growth transformation of normal B cells [14]. ROS can cause damage to DNA directly or indirectly via oxidation of the free nucleotide pool, with the accumulation of 8-oxodG, as a commonly used maker of oxidative damage. Accordingly, we found that, while virus carriage is associated with significantly increased levels of 8-oxodG in all types of EBV infected cells, irrespective of their lymphoid or epithelial cell origin (Fig. 1), EBNA1 is directly responsible for this effect as confirmed by direct correlation between the levels of EBNA1 and 8-oxodG in transfected cells expressing stable or inducible EBNA1 (Fig. 2). Most importantly, the levels of ROS, 8-oxodG, and DNA damage coordinately decreased upon downregulation of EBNA1, indicating that the oxidative stress phenotype is reversible in the absence of the viral protein.

While the induction of sustained high levels of ROS is essential for EBV-induced B-cell immortalization, possibly via the regulation of key phosphatases and miRNAs [14], specific antioxidant defenses must be activated in order to contain the antiproliferative response to oxidated DNA. Indeed, we found that EBNA1 promotes the concomitant activation of oxidated dNTPs sanitization and purging pathways, as illustrated by the upregulation of nucleoside triphosphatase MTH1 and the glycosidases OGG1 and MUTYH, respectively. MTH1 hydrolyses oxidized purines to prevent their addition onto nascent DNA chains, which may otherwise result in mispairing, mutations and cell death, while OGG1 and MUTYH excise oxidated bases from damaged DNA, which initiates both long- or and short-patch BER. OGG1 and to a minor extent MUTYH were upregulated in parallel with MTH1 in cells expressing inducible EBNA1 (Fig. 3). Furthermore, a direct correlation between the expression levels of MTH1, OGG1, MUTYH, and EBNA1 was detected in sublines of the EBV positive BL Mutu and higher levels of MHT1 were detected in western blots of four out of five EBV converted sublines of EBV-negative Burkitt lymphoma, gastric carcinoma and nasopharynx cancer while OGG1 and MUTYH exhibited a somewhat less consistent pattern of regulation (Fig. 4). These findings have interesting implications. First, they confirm the role of EBNA1 in the activation of the antioxidant response, which may reflect both a cellular adaptation to the increased levels of ROS induced by EBNA1 and an EBNA1 mediated transcriptional regulation of effector proteins. Second, the discrepancy between the consistent EBNA1-dependent upregulation of both sanitization and purging pathways in the inducible cell lines and in the originally EBV positive BL, as compared with the more variable response of the convertants, suggests that, upon prolonged selection, multiple mechanisms may be recruited to contain oxidation within levels compatible with cell survival. These may include the activation of peroxidases and other ROS quenching responses, failure to detect and respond to oxidated DNA and the activation of antiapoptotic pathways that would, alone or in different cell-specific combinations, increase tolerance to ongoing DNA damage. It is noteworthy that EBV positive BLs exhibit higher levels of ROS compared with EBV-negative tumors [30] and transcriptional activation of various components of the antioxidant pathways, including glutathione peroxidase [12] and MTH1 was detected in biopsies and cell lines derived from EBV-positive tumors (dataset: https://www.ncbi.nlm.nih.gov/geo/query/acc.cgi?acc=GSE123449). Furthermore, the genomes of EBV-positive tumors contain a greater mutational load compared with the EBV-negative variants [31], and polymorphisms of the BER effectors OGG1, APE1, and XRCC1 are associated with increased risk of NPC in the Chinese population [32], suggesting that the oxidative stress associated with infection may play an important role in carcinogenesis.

MTH1 is often upregulated in malignant cells and impressive therapeutic responses have been reported upon treatment with MTH1 inhibitors in some in vitro and in vivo tumor models [25]. However, the general validity of this finding is controversial, and the specificity and mechanism of action of the inhibitors is still actively debated [22]. We have found that EBV carrying cells are significantly more sensitive than their EBV-negative parentals to induction of apoptosis by treatment with two inhibitors of MTH1, TH588 and S-Crizotinib that differ in their overall mechanism of action and side effects [25, 26] (Fig. 5 and Table 1). In line with the role of MTH1 in the sanitization of oxidated nucleotides, the induction of apoptosis was accompanied by a highly significant increase of 8-oxodG and induction of DNA damage measured by the accumulation of γH2AX (Fig. 6). Similar to the effect on MTH1 expression, the enhanced apoptotic response was directly dependent on the expression of EBNA1 and could be reversed by downregulation of EBNA1 in the inducible cell lines BJAB-tTAE1. It is noteworthy that the BL41-E95B convertant that failed to upregulate MTH1 was particularly sensitive to the inhibitor, suggesting that the expression levels of MTH1 may be critical for the capacity of the inhibitors to tilt the oxidative balance beyond a critical threshold. The inability to kill some cancer cells by MTH1 knockdown [33], together with the resistance of some cell lines to treatment with highly specific MTH1 inhibitors [33, 34] and failure to rescue sensitive cells by overexpression of human MTH1 or the bacterial homolog that shares the enzymatic activity [35] have casted doubts on the mechanism of action of the inhibitors. The different behavior of the cell lines used in our assays point to the possibility that at least some of these failures may be explained by the type of cells used in the assays, where variations in the multifactorial control of the oxidative balance and the cell intrinsic capacity to detect and respond to oxidative DNA damage may determine the degree of dependency on MTH1. Overall, our findings corroborate previous observations linking the potency of MTH1 inhibitors to the extent of oxidative stress [35, 36], suggesting that the endogenous levels of ROS could provide a useful biomarker of sensitivity.

The dramatic increase of ROS that occurs in freshly EBV infected B-lymphocytes coincides with extensive DNA damage and cell death [14, 37], whereas ROS and DNA damage stabilize at significantly lower levels in immortalized LCLs [14], suggesting that viral latency is dependent on the activation of antioxidant defenses that reset the oxidative balance to a level compatible with cell proliferation. In line with this possibility, we found that the expression of MTH1 progressively increases in parallel with EBNA1 after infection reaching a maximum when the infected cells enter the fast proliferation phase that characterizes newly established LCLs (Fig. 7). As observed in EBV carrying cell lines, MUTYH and OGG1 were also upregulated in freshly infected cells, which is consistent with a synergistic effect of the sanitization and purging machineries in limiting DNA oxidation. It is noteworthy that, in spite of similar levels of proliferation, mitogens induced B-blasts expressed lower levels of MTH1, OGG1, and MUTYH, pointing to EBNA1 and ROS rather than the proliferative or metabolic status of the cells as the key triggering events. Further emphasizing the critical requirement of harnessing the cellular antioxidant defense in order for EBV to achieve growth transformation, we found that treatment with MTH1 inhibitors inhibited the proliferation of freshly infected B cells in a dose-dependent manner, and prevented the establishment of LCLs by promoting a dramatic increase of DNA damage.

**Fig. 8:**
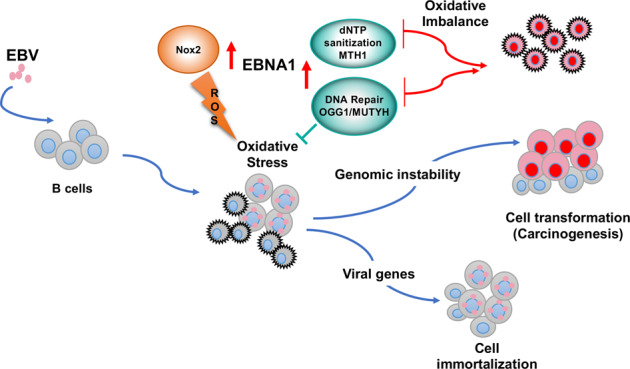
EBNA1 alters the cellular oxidative balance to promote cell immortalization and oncogenesis. EBNA1 is the only viral protein regularly expressed in all types of EBV carrying normal and malignant cells. The expression of EBNA1 is associated with a shift of the cellular redox status that correlates with transcriptional activation of the catalytic subunit of the NADPH oxidase NOX2. High levels of ROS are required for efficient expression of cellular and viral genes that sustain B-cell proliferation and immortalization, including the viral latent membrane protein LPM1. While inducing the establishment of an oxidative environment that promotes infection, EBNA1 limits the antiproliferative response triggered by oxidated DNA via activation of both the nucleotide sanitization pathway that prevents the incorporation of oxidated nucleotides, and repair pathways that purge DNA from oxidized bases. The persistent oxidative stress induced by EBNA1 is associated with genomic instability, which promotes carcinogenesis and renders EBV infected cells particularly sensitive to disturbance of the redox balance by the inhibition of antioxidant pathways

Collectively, our findings highlight a scenario where EBNA1 plays a key role in the reshaping of the cellular environment that enables EBV-induced growth transformation and generates the premalignant precursors of EBV associated cancers (Fig. 8). Via the upregulation of NOX2 and increase of intracellular ROS, EBNA1 promotes a shift in redox status that is required for efficient expression of growth transforming viral and cellular genes. The antiproliferative effect of the ensuing oxidative DNA damage is counteracted by the concomitant activation of dNTP sanitization and DNA purging machineries that, in concert with inhibition of the DNA damage and antiapoptotic responses induced by other latency gene products, including LMP1 and EBNA3A [38], contain the damage to a level compatible with cell proliferation and virus-induced immortalization. The genomic instability associated with the oxidative milieu and ongoing DNA damage may favor the selection of genetic or epigenetic changes that will eventually result in progression to full malignancy. Due to their adaptation to a highly oxidative environment, EBV carrying cells will be particularly sensitive to disturbance of the redox status pointing to the cellular antioxidant defenses as a suitable target for antiviral and anticancer therapies.

## Materials and methods

### Chemicals and antibodies

Antibodies and their suppliers were: MTH1 (1:1000, rabbit, NB100-109, NOVUS, Abingdon, Oxon, England, UK), OGG1 (1:500, rabbit, NB100-106, NOVUS), XRCC1 (1:1000, rabbit, A300-065A, Bethyl Laboratories, Montgomery, TX, USA), MUTYH (1:1000, rabbit, PA5-27855, ThermoFischer, Waltham, MA, USA), EBNA1 (1:100, rat, E1BS1H4-14111, supernatant), LMP1 (1:50, mouse, M0897, Dako, Glostrup, Denmark), BZLF1 (1:1000, mouse, SC-53904, Santa Cruz, Dallas, TX, USA), GAPDH (1:2000, mouse, CB1001, Merck Millipore, Darmstadt, Germany). Antibodies used for immunofluorescence: pH2AX (1:100, mouse, 05–636, Millipore Corporation, Billerica, MA, USA), Anti-8-oxodG (1:50, mouse, ab206461, Abcam, Cambridge, MA, USA). Chemicals and their suppliers were: TH588 (Sigma-Aldrich, SML1069, St Louis, MO, USA), (S)-Crizotinib (TOCRIS, 6025, Abingdon, Oxon, England, UK).

### Cell lines

The B lymphoma lines BJAB [39], BL41 [40], and their B95-8 convertants (BJAB-B958 and BL41-E95B), the EBV-producing marmoset B-cell line B95-8 [41], and the EBV-negative Burkitt lymphoma lines Akata [42], Mutu-30 [43], and the EBV positive Burkitt lymphoma lines Mutu I cl.148 and Mutu III cl.99 [44] were cultured in RPMI-1640 medium (R8758 Sigma-Aldrich, St Louis, MO, USA), supplemented with 10% Fetal Bovine Serum (10270, GIBCO-Invitrogen, Waltham, MA, USA), and 10 μg/ml Ciprofloxacin (Sigma-Aldrich) (complete medium). The Akata-Bx1 cell line [45] that carries a recombinant Akata EBV, where the thymidine kinase gene was replaced by a CMV immediate-early promoter-driven green fluorescent protein was cultured in complete medium supplemented with 500 μg/ml Geneticin (GIBCO-Invitrogen). The stable EBNA1 transfected BJAB-E1 cell line [46] was kept in complete medium supplemented with 500 μg/ml Geneticin (GIBCO-Invitrogen). A BJAB subline that stably expresses a tetracycline-regulated EBNA1 gene (BJAB-tTAE1) was produced by transfection of the pTRE2pur-FlagEBNA1 plasmid into the BJAB-tTA cell line [12] that carries a tet-off-regulated transactivator (kind gift of Martin Rowe, University of Birmingham, United Kingdom) followed by selection in 1 mg/mL puromycin and 500 mg/mL hygromycin B (Calbiochem). The EBV-negative gastric carcinoma line AGS and the EBV converted AGS-Bx1 cell line [47, 48] (kindly provided by Alan Chiang, Hong Kong University, Hong Kong) were cultured in Dulbecco’s Modified Eagle Medium: Nutrient Mixture F-12 (DMEM/F12) (GIBCO-Invitrogen) supplemented with 10% Fetal Bovine Serum. AGS-Bx1 was cultured in complete medium supplemented with 500 μg/ml Geneticin (GIBCO-Invitrogen). The EBV-negative nasopharyngeal carcinoma line TWO3 and the EBV converted TWO3-EBV [49, 50] (kindly provided by George Klein, Karolinska Institutet, Stockholm) were cultured in DMEM/F12 medium, supplemented with 10% Fetal Bovine Serum. TWO3-EBV was cultured in complete medium supplemented with 500 μg/ml Geneticin (GIBCO-Invitrogen).

### B-cell isolation, EBV infection, and mitogen stimulation

Blood lymphocytes were purified from Buffy coats (Blood Bank, Karolinska University Hospital, Stockholm, Sweden) by Ficoll-Paque (Lymphoprep, Axis-shield PoC AS, Oslo, Norway) density gradient centrifugation and B cells were affinity-purified using CD19 micro beads (MACS MicroBeads, Miltenyi Biotec, Bergisch Gladbach, Germany) resulting in >95% pure B-cell populations. B cells were infected with B95-8 virus containing supernatant for 1.5 h at 37 °C at a concentration of 2 × 10^6^/ml (3 × 10^5^ infectious units) and then diluted at the desired concentration without removal of the virus. For mitogen stimulation, the cultures were supplemented with heat-killed and formalin-fixed Staphylococcus aureus (1/20000 pansorbin cells, Calbiochem, Merck KGaA, Darmstadt, Germany) and 20 U/ml recombinant IL-2 (Peprotech, Rocky Hill, NJ, USA). Both B-cell cultures were maintained in RPMI-1640, supplemented with 20% fetal bovine serum and 10 μg/ml Ciprofloxacin. For cell proliferation, 10^5^ cells were cultured in 200 μl medium in 96 well plates, while for protein analysis, 10^6^ cells were cultured in 2 ml medium in 24 well plates.

### Detection of ROS

Intracellular ROS were detected by 2′,7′-dichlorodihydrofluorescein diacetate (H2DCF-DA; D6883 Sigma-Aldrich) fluorescence. Cells (1×10^6^) were incubated in 1 ml PBS containing 2 μM H2DCF-DA for 30 min at 37 °C, washed and resuspended in PBS. The fluorescence of 1 × 10^4^ cells was acquired by flow cytometry (FACSCalibur, BD Biosciences, San Jose, CA, USA) with 488 nm excitation and 530 nm emission.

### Immunofluorescence

The expression of 8-oxodG and the DNA damage marker γ-H2AX was investigated by indirect immunofluorescence. Cells (6 × 10^4^) were deposited on glass slides by cytospin centrifugation for 2 min at 600 rpm. The slides were fixed in PBS containing 4% formaldehyde for 10 min, permeabilized in PBS containing 0.5% Triton X-100 for 5 min and then blocked in PBS containing 3% bovine serum albumin for 1 h at room temperature. The slides were incubated with primary antibodies diluted in blocking buffer overnight at 4 °C followed by 3 × 10 min washes in PBS and incubation for 1 h at room temperature with the appropriate Alexa Fluor-conjugated secondary antibodies. After mounting in Vectashield-containing DAPI (Vector laboratories, Inc. Burlingame, CA, USA), images were acquired with a fluorescence microscope (Leica DM RA2, Leica Microsystems, Wetzlar, Germany) equipped with a CCD camera (C4742-95, Hamamatsu, Japan). The images were analyzed with Photoshop (Adobe Systems Inc., San Jose, CA, USA) and fluorescence was quantified with the ImageJ software.

### Immunoblotting

Cell lysates were prepared in RIPA lysis buffer (50 mM Tris-HCl pH 7.4, 150 mM NaCl, 1% Triton X-100, 1% sodium dodecyl sulfate, 0.5% sodium deoxycholate, protease and phosphatase inhibitors cocktails). Protein concentration was measured with a Protein Assay kit (Bio-Rad Laboratories, Solna, Sweden). After denaturation for 5 min at 100 °C in lithium dodecyl sulfate sample buffer (NP0008, Invitrogen, Carlsbad, CA, USA), the lysates were fractionated in precast 4–12% sodium dodecyl sulfate-polyacrylamide gel electrophoresis gradient gels (Invitrogen, Carlsbad, CA, USA) and transferred to polyvinylidene-difluoride membranes (Millipore Corporation, Billerica, MA, USA). After blocking in TBS containing 0.1% Tween-20 and 5% nonfat milk powder and incubation with primary antibody overnight at 4 °C and the appropriate HRP-conjugated secondary antibody for 1 h at room temperature, the immunocomplexes were visualized by enhanced chemiluminescence (GE Healthcare Limited, Buckinghamshire, UK).

### Reverse transcription and real-time PCR

RNA was isolated using the Quick-RNA MiniPrep kit (Zymo Research, Irvine, CA, USA) with in-column DNase treatment according to the instructions of the manufacturer. One microgram of total RNA was reverse-transcribed using SuperScript VILO cDNA Synthesis kit (Invitrogen). PCR amplification was performed with the LC FastStart DNA master SYBR green I kit in a LightCycler 1.2 instrument (Roche, Basel, Switzerland) using validated PrimePCR SYBR Green primers specific for human MTH1 (qHsaCID0038594), OGG1 (qHsaCED0043531) and MUTYH (qHsaCED0038608) (Bio-Rad Laboratories, Hercules, CA, USA). The relative level of mRNA was determined by standard curve method, using MLN51 (Metastatic lymph node 51) as housekeeping gene (5´-CAAGGAAGGTCGTGCTGGTT-3´ and 5´-ACCAGACCGGCCACCAT-3´).

### Propidium iodide/Annexin-V staining

Cells were harvested into tubes, washed with cold PBS for two times. Each sample (1 × 10^5^ cells) was resuspended in 100 µl of 1 × Annexin-V binding buffer (51-66121E, BD Bioscience, San Diego, CA, USA). After addition of Annexin V-FITC (51–65874 × , BD Bioscience) and PI (51–66211E, BD Bioscience) the tubes were incubated for 15 min in the dark followed by addition of 400 µl of binding buffer. Cell death and apoptosis was detected by flow cytometry within 1 h.

### ^3^H-Thy incorporation

At the indicated time points, 1 μCi ^3^H-thymidine was added to each well and the plates were incubated at 37 °C in 5% CO_2_ for 16 h. The cells were harvested on a glass fibre filter and radioactivity was measured in a liquid scintillation counter. All data represent the mean incorporation of five parallel wells.

## Supplementary information

Supplementary information.
