# Supplementary material for: The Epstein–Barr virus nuclear antigen-1 upregulates the cellular antioxidant defense to enable B-cell growth transformation and immortalization
Source: Oncogene. 2019 Sep 11;39(3):603–16. doi: 10.1038/s41388-019-1003-3 (PMC6962091; doi:10.1038/s41388-019-1003-3)
Supplement: Supplementary file 1 — Supplementary information. [file 41388_2019_1003_MOESM1_ESM.pdf]

Supplementary information

Fig. S1. Time course of ROS accumulation in BJAB-tTAE1 cells cultured in the presence or absence of doxycycline. The levels of intracellular ROS were quantified by H2DCF-DA fluorescence in BJAB-tTAE1 cells cultured for the indicated times in the presence or absence of doxycycline.

Fig. S2. The induction of MTH1 is dependent on EBNA1 expression. Representative western blots illustrating the expression of MTH1 in BJAB-tTA cells stably transfected with the empty -tTA transactivator plasmid cultured with or without doxycycline. GAPDH was used as loading control.

Fig. S3. TH588 treatment does not induce apoptosis in EBNA1 negative cells. BJAB-tTA cells cultured with or without doxycycline were treated for 48 hrs with 10 μM of the MTH1 inhibitor TH588. Apoptosis was measured by Annexin-V and PI staining. Mean ± SE of two experiments.

Fig. S4. Treatment with TH588 does not affect the expression of viral antigens in EBV positive cells. The cells were treated with 10 μM of TH588 for the indicated times and western blots of total cell lysates were probed with the indicated antibodies. One representative western blot out of three is shown in the figure.

Fig. S1

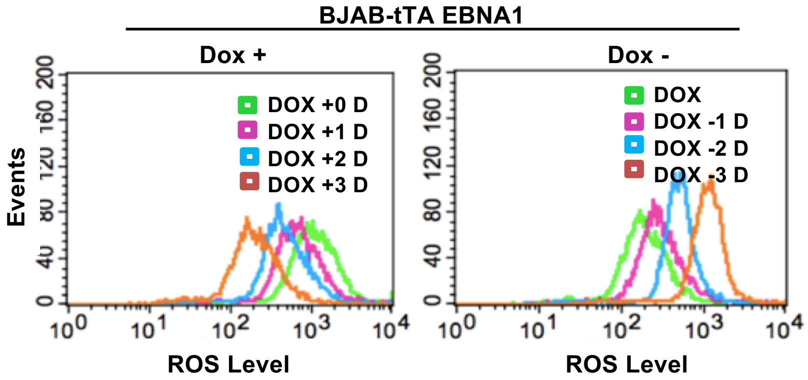

BJAB-tTA EBNA1
Dox +
Dox -
DOX +0 D
DOX +1 D
DOX +2 D
DOX +3 D
DOX
DOX -1 D
DOX -2 D
DOX -3 D
Events
ROS Level
ROS Level

Fig. S2

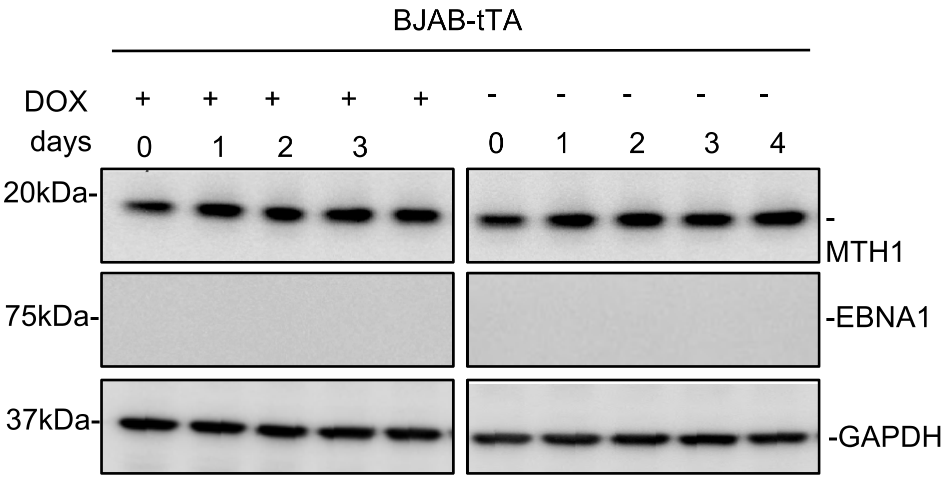
BJAB-tTA
DOX + + + + + - - - - -
days 0 1 2 3 0 1 2 3 4
20kDa-
75kDa-
37kDa-
-MTH1
-EBNA1
-GAPDH

Fig. S3

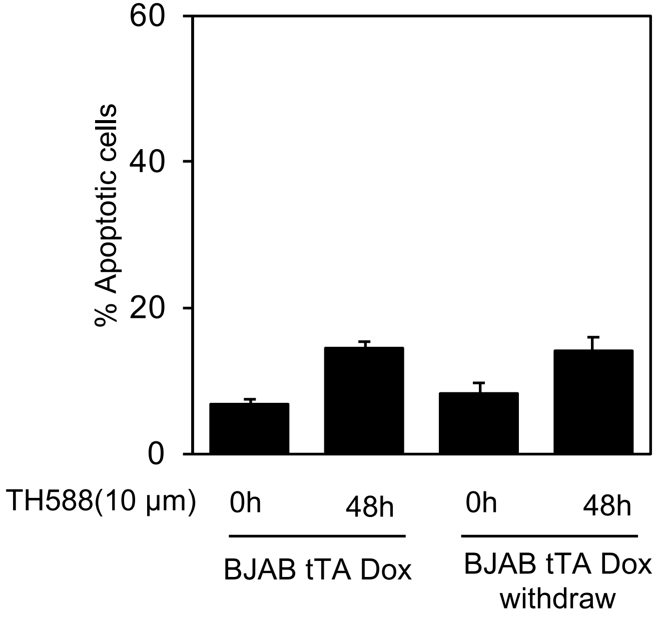

60
40
20
0
% Apoptotic cells
TH588(10 μm)
0h
48h
0h
48h
BJAB tTA Dox
BJAB tTA Dox withdraw

Fig. S4

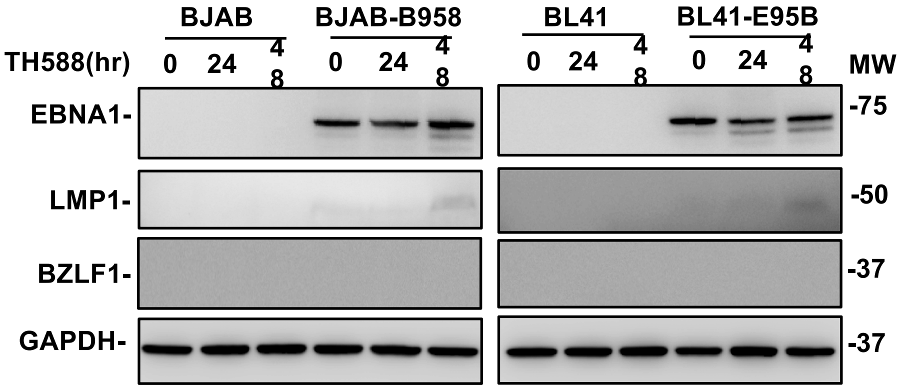

BJAB
BJAB-B958
BL41
BL41-E95B
TH588(hr)
0
24
4 8
0
24
4 8
0
24
4 8
0
24
4 8
MW
EBNA1-
-75
LMP1-
-50
BZLF1-
-37
GAPDH-
-37
